# Knowledge and Attitudes of Healthcare Professionals Regarding Disabilities in Eastern India

**DOI:** 10.7759/cureus.75267

**Published:** 2024-12-07

**Authors:** Deepak Kumar, Rajan Kumar, Bijit Biswas, Labani Biswas, Satya Ranjan Patra

**Affiliations:** 1 Physical Medicine and Rehabilitation, All India Institute of Medical Sciences, Deoghar, Deoghar, IND; 2 Pediatrics, All India Institute of Medical Sciences, Deoghar, Deoghar, IND; 3 Community and Family Medicine, All India Institute of Medical Sciences, Deoghar, Deoghar, IND; 4 General Surgery, All India Institute of Medical Sciences, Deoghar, Deoghar, IND

**Keywords:** attitude, disabilities, disabled persons, government schemes, healthcare personnel, india, knowledge

## Abstract

Background: Globally, a substantial portion of the population lives with significant disabilities. Despite advancements, individuals with disabilities continue to experience poorer health outcomes, often due to inadequate knowledge and attitudes among healthcare providers. This study aimed to evaluate the knowledge and attitudes of healthcare professionals regarding disabilities in a tertiary care setting.

Methodology: A cross-sectional survey was conducted among healthcare professionals using an online questionnaire. The survey assessed their knowledge of disability-related laws, attitudes regarding inclusion, and perceptions of responsibilities toward persons with disabilities (PwD).

Results: The study included 126 (54.5%) female participants, of which nurses comprised 146 (63.2%), followed by doctors (n=66, 28.6%) and support staff (n=19, 8.2%). Gender differences showed males had greater awareness of Government of India schemes (n=12, 11.4% in males vs. n=2, 1.6% in females, p = 0.002), while female participants demonstrated better understanding of healthcare responsibilities (p = 0.043) and stronger support for free healthcare for PwD (n=88, 69.8% vs. n=60, 57.1%, p = 0.045]. Doctors had higher knowledge of disability certification (n=18, 27.3%) than nurses (n=24, 14.4%) (p = 0.023), while 141 (85.5%) nurses and support staff supported equal social opportunities compared to 34 (51.5%) doctors (p = 0.001). The average knowledge and attitude scores were 5.1±1.6 and 7.6±2.6, respectively, with a moderate positive correlation between the two (r = 0.388, p < 0.001).

Conclusion: Healthcare personnel demonstrated satisfactory knowledge and attitudes toward disabilities, with nursing officers excelling in attitudes and doctors in certification knowledge. Gaps in legal and scheme awareness underscore the need for targeted training to enhance inclusion.

## Introduction

Globally, over 1.3 billion people, or 16% of the world's population, live with a significant disability, with children (0-14 years) comprising 5.8% of the total global population with disabilities. This number continues to rise, driven by increasing noncommunicable diseases and improved longevity [1]. Of this, 15.3% have moderate-to-severe disabilities, and 2.9% experience severe disabilities [2]. The World Health Organization (WHO) defines disability as any physical or mental impairment that limits an individual’s ability to perform specific activities and engage with their environment [1-3].

Despite global efforts, persons with disabilities (PwDs) consistently experience poorer health outcomes compared to those without disabilities, irrespective of a country's economic status [4,5]. This disparity is partly attributed to the insufficient focus on their unique health needs, coupled with a lack of expertise among healthcare providers and widespread misconceptions about disabilities within the healthcare sector [4,6]. The knowledge, attitudes, and perceptions of healthcare professionals significantly influence the quality of care provided, often dictating whether it is equitable and inclusive [4,5,7].

In India, where the prevalence of disability is considerable, addressing the attitudes and understanding of healthcare professionals is critical to improving care delivery [8,9]. However, evidence on this subject remains limited, particularly in Eastern India, where healthcare challenges are compounded by resource constraints. This study seeks to assess the knowledge and attitudes of tertiary care healthcare professionals regarding disabilities through an online cross-sectional survey. The findings aim to inform strategies for capacity building and sensitization programs to bridge existing gaps and promote disability-inclusive healthcare practices.

## Materials and methods

This cross-sectional survey was conducted at the All India Institute of Medical Sciences (AIIMS), Deoghar, Jharkhand, India, between December 2023 and April 2024. AIIMS Deoghar serves as a tertiary care facility providing comprehensive healthcare services, including medical, surgical, diagnostic, outpatient, rehabilitative, and support services, to a population of approximately 32,988,134. The institute is staffed with a diverse range of clinical and non-clinical personnel and adopts a multidisciplinary approach to healthcare delivery.

Sample size

A total of 542 healthcare professionals were directly involved in patient care, either in outpatient (OPD) or inpatient (IPD) settings, at the study institute during the data collection period. This included 115 doctors, 341 nurses, and 86 other support staff, such as attendants and cleaning personnel. The sample size for the study was determined using Taro Yamane’s formula: n=N/[1+N(e^2^)], where N represents the total population in the sampling frame (542), and e is the margin of error (0.05). Based on this calculation, the minimum required sample size was 230 participants.

Data collection

Data were collected using convenience sampling through an online survey via Google Forms (Google LLC, Mountain View, California, United States) that recorded demographic details, including gender, occupation, and the presence of any physical disabilities among participants or their family members (Appendices). The questionnaire also assessed knowledge and attitudes regarding disability. Knowledge was evaluated across three domains: the Rights of PwD (RPwD) Act 2016, the legal obligations of society, and the responsibilities of healthcare professionals. Participants rated their familiarity with these areas on a scale ranging from 'Not familiar at all' (0) to 'Very familiar' (2). Knowledge scores were calculated based on correct responses to eight knowledge-related items, with higher scores indicating greater knowledge.

Attitudes were assessed using a 10-item questionnaire. Positive responses were scored as 1, while the question on discomfort was inversely scored. The total attitude score was derived by summing all item scores, with higher scores reflecting more positive attitudes. The reliability of the knowledge and attitude questionnaires was evaluated using Cronbach’s alpha, yielding values of 0.702 and 0.806, respectively, indicating acceptable and good reliability.

Ethical considerations

Prior to initiating the study, ethical approval was obtained from the Ethics Committee of AIIMS Deoghar (approval number: 2022-76 IND-02). The study objectives were clearly detailed at the beginning of the administered survey, and online informed consent was secured before participant inclusion. Confidentiality was ensured by anonymizing all data, which were used solely for research purposes. The study adhered strictly to the ethical principles outlined in the Declaration of Helsinki.

Statistical analysis

Data analysis was performed using JAMOVI (version 2.3.26; Retrieved from https://www.jamovi.org), an open-source statistical software. Comparative analyses of individual knowledge and attitude items were conducted across gender and occupation using chi-square tests. Pearson’s correlation was used to examine the relationship between background characteristics, knowledge scores, and attitude scores, with the strength of the association expressed through correlation coefficients (r). Statistical significance was determined at a p-value of <0.05.

## Results

Female participants constituted 54.5% (n=126) of the sample population. The majority of the study participants were nurses (n=146, 63.2%), followed by doctors (n=66, 28.6%) and other staff (n=19, 8.2%). Approximately a quarter (n=66, 28.6%) of the participants reported having friends or family members with disabilities, while two of them identified as having disabilities themselves. When examining gender disparities concerning various knowledge areas, disparities emerged in awareness of various Government of India (GOI) schemes. For instance, knowledge about schemes like Vikaas-Day Care Scheme For PwD Children [10] was significantly higher among male participants (n=12, 11.4%) compared to female participants (n=2, 1.6%), with a p-value of 0.002. Similarly, awareness of Unique Disability Identification (UDID) [11] was notably different between male participants (n=8, 7.6%) and female participants (n=28, 22.2%), with a p-value of 0.002. Additionally, female participants exhibited greater awareness of healthcare worker responsibilities regarding providing accessibility for PwD in our society (p = 0.043). Regarding attitudes, a larger proportion of female participants (n=88, 69.8%) believed that PwD should receive the best healthcare facilities free of charge, in contrast to male participants (n=60, 57.1%) (p-value=0.045) (Table 1).

**Table 1:** Distribution of the knowledge and attitude regarding disability as per gender of study participants (N=231) #Chi square test; *significant p-value RPwD: rights of person with disability; PwD: person with disabilities; ADIP: Assistance to Disabled Persons for Purchase/Fitting of Aids/Appliances

Variable	Total participants, n (%)	Male participants (n=105), n (%)	Female participants (n=126), n (%)	p-value^#^
Knowledge Items				
Are you aware of Government of India schemes for PwD?	-	-	-	-
Vikaas-Day Care Scheme For Person with Disability Children [10]	14 (6.1)	12 (11.4)	2 (1.6)	0.002^*^
Unique Disability Identification [11]	36 (15.6)	8 (7.6)	28 (22.2)	0.002^*^
Disability Certification for 21 diseases [12]	42 (18.2)	22 (21.0)	20 (15.9)	0.319
ADIP scheme [13]	38 (16.5)	20 (19.0)	18 (14.3)	0.331
How familiar are you with the RPwD Act 2016 [14]?	-	-	-	-
Not Familiar at all	87 (37.7)	43 (41.0)	44 (34.9)	0.167
Somewhat Familiar	124 (53.7)	50 (47.6)	74 (58.7)	-
Very Familiar	20 (8.7)	12 (11.4)	8 (6.3)	-
How familiar are you with society’s legal obligation to provide accessibility to PwDs?	-	-	-	-
Not Familiar at all	38 (16.5)	14 (13.3)	24 (19.0)	0.098
Somewhat Familiar	149 (64.5)	65 (61.9)	84 (66.7)	-
Very Familiar	44 (19.0)	26 (24.8)	18 (14.3)	-
How familiar are you with your responsibilities as a healthcare worker for providing accessibility for PwDs in our society?	-	-	-	-
Not Familiar at all	10 (4.3)	8 (7.6)	2 (1.6)	0.043^*^
Somewhat Familiar	96 (41.6)	38 (36.2)	58 (46.0)	-
Very Familiar	125 (54.1)	59 (56.2)	66 (52.4)	-
Do you think this Institute's campus follows the guidelines of Saugamya Bharat/Accessible India?	-	-	-	-
Yes	108 (46.8)	44 (41.9)	64 (50.8)	0.178
No	123 (53.2)	61 (58.1)	62 (49.2)	-
Attitude Items				
Do you feel PwDs have equity in social opportunities?	-	-	-	-
Yes	175 (75.8)	75 (71.4)	100 (79.4)	0.161
No	56 (24.2)	30 (28.6)	26 (20.6)	-
Do you think prompt awareness and disability-related issues should be discussed in our society?	-	-	-	-
Yes	225 (97.4)	101 (96.2)	124 (98.4)	0.290
No	6 (2.6)	4 (3.8)	2 (1.6)	-
Do you feel pity on seeing PwDs?	-	-	-	-
Yes	120 (51.9)	50 (47.6)	70 (55.6)	0.229
No	111 (48.1)	55 (52.4)	56 (44.4)	-
Do you feel awkward or embarrassed on coming across PwDs?	-	-	-	-
Yes	26 (11.3)	14 (13.3)	12 (9.5)	0.362
No	205 (88.7)	91 (86.7)	114 (90.5)	-
What improvements does our society need for PwDs?	-	-	-	-
Accessibility in infrastructure	168 (72.7)	82 (78.1)	86 (68.3)	0.094
Accessibility in transport system	150 (64.9)	74 (70.5)	76 (60.3)	0.107
Accessibility in communication	144 (62.3)	62 (59.0)	82 (65.1)	0.346
Best healthcare facility free of cost	148 (64.1)	60 (57.1)	88 (69.8)	0.045^*^
Scholarship on education	142 (61.5)	64 (61.0)	78 (61.9)	0.882
Supply of prosthetic appliances, free cloth items	154 (66.7)	66 (62.9)	88 (69.8)	0.262

When examining occupational differences in knowledge, it was found that doctors (n=18, 27.3%) exhibited higher awareness regarding disability certification for 21 diseases compared to other professions (n=24, 14.5%) (p = 0.023). In exploring occupational variations in attitudes, a larger proportion of nurses and allied staff (n=141, 85.5%) expressed the belief that PwDs should have equal access to social opportunities compared to doctors (n=34, 51.5%) (p = 0.001). Moreover, doctors showed significantly stronger support for societal improvements for disabled individuals in four out of the six measured aspects compared to other healthcare personnel (Table 2).

**Table 2:** Distribution of the knowledge and attitude regarding disability as per occupation of study participants (N=231) #Chi square test; *significant p-value RPwD: rights of person with disability; PwD: person with disabilities; ADIP: Assistance to Persons with Disabilities for Purchase/Fitting of Aids/Appliances

Variable	Total participants, n (%)	Doctors (n=66), n (%)	Nursing & Allied Staff (n=165), n (%)	p-value^#^
Knowledge Items				
Are you aware of Government of India schemes for PwDs?	-	-	-	-
Vikaas-Day Care Scheme For Person with Disability Children [10]	14 (6.1)	6 (9.1)	8 (4.8)	0.222
Unique Disability Identification [11]	36 (15.6)	14 (21.2)	22 (13.3)	0.136
Disability Certification for 21 diseases [12]	42 (18.2)	18 (27.3)	24 (14.5)	0.023^*^
ADIP scheme [13]	38 (16.5)	10 (15.2)	28 (17.0)	0.736
How familiar are you with RPwD Act 2016 [14]?	-	-	-	-
Not Familiar at all	87 (37.7)	24 (36.4)	63 (38.2)	0.964
Somewhat Familiar	124 (53.7)	36 (54.5)	88 (53.3)	
Very Familiar	20 (8.7)	6 (9.1)	14 (8.5)	-
How familiar are you with society’s legal obligation to provide accessibility to PwD?	-	-	-	-
Not Familiar at all	38 (16.5)	8 (12.1)	30 (18.2)	0.478
Somewhat Familiar	149 (64.5)	46 (69.7)	103 (62.4)	-
Very Familiar	44 (19.0)	12 (18.2)	32 (19.4)	-
How familiar are you with your responsibilities as a healthcare worker for providing accessibility for PwDs in our society?	-	-	-	-
Not Familiar at all	10 (4.3)	4 (6.1)	6 (3.6)	0.468
Somewhat Familiar	96 (41.6)	30 (45.5)	66 (40.0)	-
Very Familiar	125 (54.1)	32 (48.5)	93 (56.4)	-
Do you think this institute's campus follows the guidelines of Saugamya Bharat/Accessible India?	-	-	-	-
Yes	108 (46.8)	26 (39.4)	82 (49.7)	0.156
No	123 (53.2)	40 (60.6)	83 (50.3)	-
Attitude Items				
Do you feel PwDs have equity in social opportunities?	-	-	-	-
Yes	175 (75.8)	34 (51.5)	141 (85.5)	<0.001^*^
No	56 (24.2)	32 (48.5)	24 (14.5)	
Do you think prompt awareness and disability-related issues should be discussed in our society?	-	-	-	-
Yes	225 (97.4)	64 (97.0)	161 (97.6)	0.794
No	6 (2.6)	2 (3.0)	4 (2.4)	-
Do you feel pity on seeing PwDs?	-	-	-	-
Yes	120 (51.9)	40 (60.6)	80 (48.5)	0.096
No	111 (48.1)	26 (39.4)	85 (51.5)	-
Do you feel awkward or embarrassed on coming across PwDs?	-	-	-	-
Yes	26 (11.3)	10 (15.2)	16 (9.7)	0.236
No	205 (88.7)	56 (84.8)	149 (90.3)	-
What improvements does our society need for PwDs?	-	-	-	--
Accessibility in infrastructure	168 (72.7)	58 (87.9)	110 (66.7)	0.001^*^
Accessibility in transport system	150 (64.9)	54 (81.8)	96 (58.2)	0.001^*^
Accessibility in communication	144 (62.3)	46 (69.7)	98 (59.4)	0.144
Best healthcare facility free of cost	148 (64.1)	42 (63.6)	106 (64.2)	0.931
Scholarship on education	142 (61.5)	48 (72.7)	94 (57.0)	0.026^*^
Supply of prosthetic appliances, free cloth items	154 (66.7)	52 (78.8)	102 (61.8)	0.013^*^

**Figure 1:**
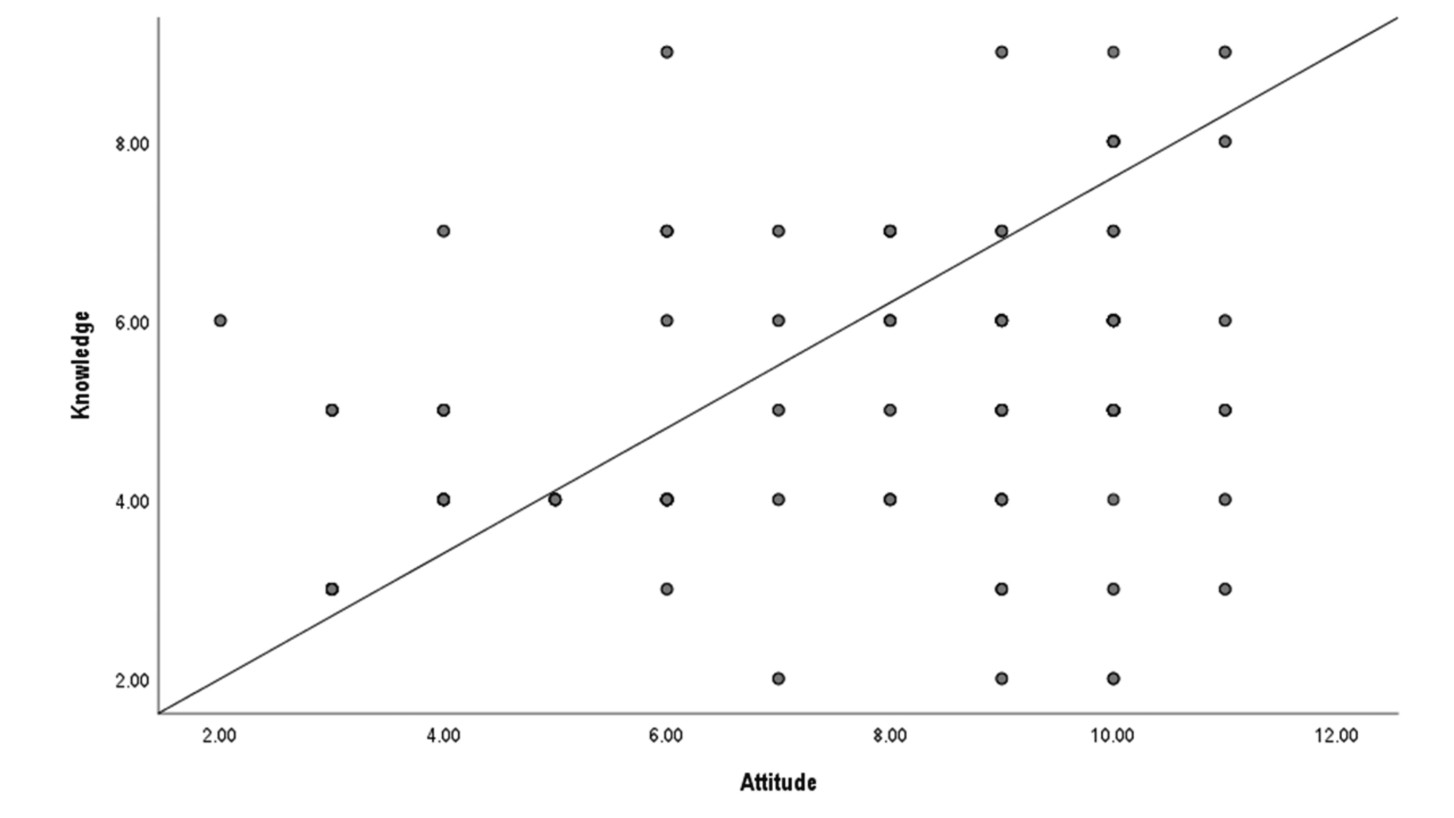
Scatter plot showing correlation between knowledge and attitude scores regarding disability (N=231)

The average knowledge score was 5.1±1.6, and the average attitude score stood at 7.6±2.6. A moderate correlation was observed between knowledge and attitude scores (r = 0.388, p = <0.001). Female participants (r = 0.155, p = 0.019) and individuals with friends or relatives having disabilities (r = 0.166, p = 0.014) were more likely to possess higher levels of knowledge. Meanwhile, doctors (r = 0.22, p = 0.001) and those with friends or relatives with disabilities (r = 0.22, p = 0.001) were more inclined toward a positive attitude. (Figure 1 and Table 3).

**Table 3:** Correlation between background characteristics and knowledge and attitude regarding disability (N=231) #Pearson’s correlation test; *significant p-value PwD: person with disabilities

Variable	Knowledge regarding Disability (Correlation Co-efficient)	p-value^#^	Attitude regarding Disability (Correlation Co-efficient)	p-value^#^
Gender (Female)	0.154	0.019^*^	0.016	0.814
Occupation (Doctor)	0.010	0.875	0.220	0.001^*^
Having a PwD friend or relative (Yes)	0.162	0.014^*^	0.228	<0.001^*^

## Discussion

This cross-sectional survey provides critical insights into the knowledge and attitudes of healthcare professionals regarding disabilities, highlighting disparities and areas for improvement. Nurses constituted the largest group of participants, followed by doctors and support staff, with female participants slightly outnumbering males. Approximately one-fourth of the participants had friends or family members with disabilities, and two individuals self-identified as having a disability. Unlike previous studies, such as those by Khan et al. [15], which did not report the inclusion of healthcare professionals with disabilities, this study explicitly documented their participation, ensuring a more inclusive and nuanced perspective.

The study revealed significant gender and occupational differences in knowledge and attitudes. Female participants demonstrated greater awareness of healthcare responsibilities such as ensuring accessibility and equitable care for PwDs, while male participants exhibited superior knowledge of government initiatives like the UDID scheme. Doctors displayed greater knowledge of disability certification processes, likely due to their specialized training, while nurses and allied staff expressed stronger support for equal social opportunities for persons with disabilities. These findings align with research by Tervo et al., which indicated that females and individuals with experience in disability-related fields held more positive attitudes [16]. Additionally, the current study found a significant correlation between personal connections to PwDs and higher knowledge and attitude scores, contrasting with studies by Şimşek et al. [17] and Chadd et al. [18], which reported no significant relationships in similar contexts. This highlights the potential influence of social interactions in shaping perceptions, particularly in the Indian healthcare setting.

While these results underscore positive attitudes among healthcare professionals, they also reveal notable gaps in knowledge. For instance, awareness of the RPwD Act and related government schemes was inconsistent. Similar findings were observed in a systematic review by Satchidanand et al. [19], which reported that healthcare professionals often exhibited positive attitudes toward disability, but pockets of unfavorable views persisted, as seen in studies by Khan et al. [15], Kim et al. [20] and Tervo et al. [16]. This underscores the complexity of addressing biases and ensuring uniform understanding among healthcare providers. Educational interventions that focus on improving knowledge of legal frameworks, patient rights, and best practices for disability-inclusive care are essential. Enhanced professional and social interactions with persons with disabilities, as supported by Tervo et al. [16], can further improve attitudes and understanding, reinforcing the importance of integrating disability-related training into healthcare curricula.

The study also highlighted systemic barriers faced by persons with disabilities, including stigma, inadequate infrastructure, financial challenges, and limited access to rehabilitation services. These barriers are particularly pronounced in areas like sexual and reproductive health (SRH), where stigma and resource limitations exacerbate exclusion [21,22]. Ebere et al. similarly emphasized that societal stigma and poor infrastructure hinder access to essential services [23]. Addressing these issues requires a multifaceted approach, including significant investments in accessibility, infrastructure, and inclusive practices. Community-based rehabilitation (CBR) and quality management systems (QMS), as demonstrated by Derendorf et al. [24] and others [25,26], can significantly enhance the quality of life and social integration of persons with disabilities.

The study has several limitations. Firstly, its focus on healthcare professionals excludes public attitudes, which are crucial for shaping inclusive policies and community interventions. Secondly, the single-center design in a tertiary care facility in Jharkhand limits generalizability, as attitudes and knowledge may vary across regions and healthcare levels. Thirdly, reliance on an online survey introduces potential selection bias by excluding those with limited digital access. Lastly, the study does not account for prior training or experiences related to disability care, which may influence participants' responses. Future multi-center, mixed-method studies addressing these gaps are needed for a more comprehensive understanding.

## Conclusions

Healthcare personnel in this study demonstrated satisfactory levels of knowledge and attitude regarding individuals with disabilities. Female participants, particularly nursing officers with prior personal or professional contact with individuals with disabilities, exhibited higher levels of understanding and more positive attitudes. Doctors showed better knowledge of disability certification, while nurses and allied staff were more supportive of social equity for persons with disabilities. Gaps were identified, particularly in awareness of certain government schemes and legal responsibilities, indicating areas where targeted educational interventions might be beneficial. Expanding disability-focused training programs and outreach initiatives across all clinical personnel could further enhance inclusion and acceptance.

## Appendices

Questionnaire

**Table 4:** Study Questionnaire

Questions	Answer
Gender	Male / Female
Occupation	Doctor / Nurse / Allied Staff
Do you have any family members with disabilities?	Yes / No
Knowledge regarding disabilities	-
Are you aware of Government of India schemes for Persons with disabilities?	-
Vikaas-Day Care Scheme For Person with Disability Children	Yes / No
Unique Disability Identification	Yes / No
Disability Certification for 21 diseases	Yes / No
Assistance to Persons with Disabilities for Purchase/Fitting of Aids /Appliances (ADIP) scheme	Yes / No
How familiar are you with the Rights of Persons with Disabilities (RPwD) Act 2016?	Not Familiar at all / Somewhat Familiar / Very Familiar
How familiar are you with society’s legal obligation to provide accessibility to Persons with disabilities?	Not Familiar at all / Somewhat Familiar / Very Familiar
How familiar are you with your responsibilities as a health care worker for providing accessibility for Persons with disabilities in our society?	Not Familiar at all/ Somewhat Familiar / Very Familiar
Attitude regarding disabilities	-
Do you think this institute's campus is according to Saugamya Bharat/Accessible India?	Yes / No
Do you feel Persons with disabilities have equity in social opportunities?	Yes / No
Do you think prompt awareness and disability-related issues should be discussed in our society?	Yes / No
Do you feel pity to see Persons with disabilities?	Yes / No
Do you feel awkward or embarrassed on coming across Persons with disabilities?	Yes / No
What improvements does our society need for persons with disabilities?	-
Accessibility in infrastructure	Yes / No
Accessibility in Transport system	Yes / No
Accessibility in Communication	Yes / No
Best health care facility free of cost	Yes / No
Scholarship on education	Yes / No
Supply of prosthetic appliances, free cloth items	Yes / No
